# *In Vitro* Antiparasitic and Apoptotic Effects of Antimony Sulfide Nanoparticles on *Leishmania infantum*

**DOI:** 10.1155/2012/756568

**Published:** 2012-06-26

**Authors:** Saied Soflaei, Abdolhossein Dalimi, Fatemeh Ghaffarifar, Mojtaba Shakibaie, Ahmad Reza Shahverdi, Mohsen Shafiepour

**Affiliations:** ^1^Department of Parasitology, Medical Sciences Faculty, Tarbiat Modares University, P.O. Box 14115-111, Tehran, Iran; ^2^Pharmaceutical Biotechnology Department, Faculty of Pharmacy, Kerman University of Medical Sciences, Kerman, Iran; ^3^Pharmaceutical Sciences Research Centre, Faculty of Pharmacy, Tehran University of Medical Sciences, P.O. Box 14115-331, Tehran 14117-13116, Iran; ^4^Intramedicine Department, Afzalipour School of Medicin, Kerman University of Medical Sciences, Kerman, Iran

## Abstract

Visceral leishmaniasis is one of the most important sever diseases in tropical and subtropical countries. In the present study the effects of antimony sulfide nanoparticles on *Leishmania infantum in vitro* were evaluated. Antimony sulfide NPs (Sb_2_S_5_) were synthesized by biological method from *Serratia marcescens* bacteria. Then the cytotoxicity effects of different concentrations (5, 10, 25, 50, and 100 **μ**g/mL) of this nanoparticle were assessed on promastigote and amastigote stages of *L. infantum*. MTT method was used for verification results of promastigote assay. Finally, the percentages of apoptotic, necrotic, and viable cells were determined by flow cytometry. The results indicated the positive effectiveness of antimony sulfide NPs on proliferation of promastigote form. The IC_50_ (50% inhibitory concentration) of antimony sulfide NPs on promastigotes was calculated 50 **μ**g/mL. The cytotoxicity effect was dose-dependent means by increasing the concentration of antimony sulfide NPs, the cytotoxicity curve was raised and the viability curve of the parasite dropped simultaneously. Moreover, the IC_50_ of antimony sulfide NPs on amastigote stage was calculated 25 **μ**g/mL. On the other hand, however, antimony sulfide NPs have a low cytotoxicity effect on uninfected macrophages but it can induce apoptosis in promastigote stage at 3 of 4 concentrations.

## 1. Introduction 

Leishmaniasis is considered as one of the most important tropical diseases with worldwide distribution [1]. The disease is reported in 88 countries around the world, and its prevalence is estimated to be approximately 12 million annually and about 350 million people are at the risk of the disease [1, 2]. About 90% of cases of cutaneous leishmaniasis are found in Brazil, Afghanistan, Iran, Peru, Saudi Arabia, and Syria, and about 90% cases of visceral leishmaniasis are reported in Bangladesh, Brazil, Nepal, India, and Sudan [3, 4]. Visceral leishmaniasis (Kala-azar) is characterized by the presence of fever, splenomegaly, hepatomegaly, swollen lymph nodes, and weight loss that depends on the pathogenicity of *Leishmania* species and the host immune response against parasite [5, 6]. About 90% of the cases of this disease may lead to death if it is left without any treatment. Leishmaniasis coinfection with HIV and other immunosuppression is becoming another serious problem, therefore the treatment methods mostly focus on induction of immune responses [5]. Pentavalent antimonials are a group of compounds used for the treatment of leishmaniasis. The compounds currently available for clinical use are sodium stibogluconate (Pentostam) and meglumine antimonate (Glucantime). In systemic therapy of leishmaniasis these drugs are used alone or in combination with other compounds [7–15]. The current drugs is not so much suitable due to resistance reported, high toxicity, various side effects and so forth. So, new therapeutic antileishmanial strategies are urgently required [5, 16].

Nanomedicine is the medical application of nanotechnology. Nanomedical approaches to drug delivery center on developing nanoscale particles [17]. Up to now various nanoparticle compounds have been introduced against leishmaniasis [18–27]. In the present study we evaluated the effects of antimony sulfide nanoparticles on *Leishmania infantum in vitro*. 

## 2. Materials and Methods

### 2.1. Drug Preparation

Antimony sulfide nanoparticle was synthesized by intracellular biological methods from nonpigmented ** **by using of the *Serratia marcescens* bacterial isolate from the Caspian Sea in northern of Iran with the size less than 35 nm according to Bahrami et al. [28] in the Department of Pharmaceutical Biotechnology and Pharmaceutical Sciences Research Centre, Faculty of Pharmacy, Tehran University of medical sciences. 

### 2.2. Parasites

*Leishmania infantum* MON-1 (MHOM/TN/80/IPT1) was provided from Pasteur Institute of Iran. Promastigotes were cultured in RPMI1640 medium supplemented with 10% fetal calf serum (FCS) and antibiotics (100 IU/mL of penicillin and 100 *μ*g/mL of streptomycin). The culture was maintained in 24°C for promastigote proliferation. The parasites were transferred weekly from previous culture into new medium.

### 2.3. Drug Assessment

The interaction of antimony sulfide NPs directly and promastigotes was studied. After proliferation of the parasites, 100 *μ*L of promastigotes (2 × 10^6^ cell/mL) was seeded in 24-well plate containing 100 *μ*L of RPMI1640 medium and treated with serial dilutions of the antimony sulfide NPs (5, 10, 25, 50, and 100 *μ*g/mL) for 24, 48, and 72 hours. After incubation, the antileishmanial activity of antimony sulfide NPs was evaluated by direct counting of parasites. These data were analyzed by Graph pad Prism version 5.04 software.

### 2.4. MTT Test

Briefly, 100 *μ*L of promastigotes (2 × 10^6^ cells/mL) was cultured separately in 96-well microplates containing 100 *μ*L of RPMI1640 medium supplemented with 20%  FCS. These cultures were repeated at least three times in triplicate wells. 200 *μ*l of promastigotes were cultured as control group. 200 *μ*L/well PBS was added around well of plates to prevent the evaporation of well contents. The cells were incubated in presence seven dilutions of antimony sulfide NPs at 24 ± 1°C for 72 hours and then 20 *μ*L of MTT solution was added into each of wells. Plates were incubated again at 24°C for 4 hours and then centrifuged at 1000 g for 10 minutes. Supernatant was aspirated gently and discarded. 100 *μ*L DMSO was added to each of the wells and finally the absorbance of these plates was measured by the ELISA reader system in at 540 nm.

### 2.5. Macrophage Cytotoxicity Measurement

Inbred male BALB/c mice were prepared from Razi Institute of Iran. The effect of antimony sulfide NPs on macrophages of infected and uninfected mice was evaluated. In this regard, 7 mL of RPMI medium (sigma) was injected into peritoneumand macrophages were collected. Then the number of live macrophages was counted. 100 *μ*L of macrophages with 100 *μ*L RPMI1640 medium were seeded in exposure to seven dilutions of antimony sulfide NPs. These cultures were maintained at 37°C in the presence of 5% CO_2_ for 24, 48, and 72 hours. The experiment was terminated by direct counting. Cytotoxic effect of antimony sulfide NPs on macrophage was evaluated and compared with control cultures. 

### 2.6. Intracellular Amastigote Assay

Peritoneal cavity macrophages of BALB/c were seeded in 24-well plates and incubated at 37°C with 5% CO_2_ for 24 hours for differentiation. The cells were infected with promastigotes of stationary growth phase at a parasite/macrophage ratio of 10 : 1. Drug susceptibilities of intracellular amastigotes were assessed with the method previously described by Tada et al. [29]. The culture was incubated at 37°C in the presence of 5% CO_2_ for 24 hours until promastigotes were phagocyte by macrophages. After incubation, each well of the plates was washed with 1-2 mL PBS to remove the extracellular promastigotes. Then infected macrophages were separated from the plates by cold method (10–15 minutes on ice pieces). Then 5 *μ*L of these cells were stained by Giemsa method. The percentage of infected cells and the number of amastigotes in each cell was microscopically assessed. After that 100 *μ*L of these cells were transferred into new plate and were incubated with seven dilutions of antimony sulfide NPs at 37°C with 5% CO_2_ for 24, 48, and 72 hours. Finally, the plates were incubated on ice pieces for 10–15 minutes. The percentage of infection and IC_50_ was calculated through examination of 200 macrophages and the number of amastigotes in every single cell. The results were expressed as the infection index, which is reflecting of drug effect in prevention of infection. 

### 2.7. Promastigote Apoptosis Assessment

At first 2 × 10^6 ^cells/ml of promastigotes were treated with various dilutions (10, 25, 50, 100 *μ*g/mL) of antimony sulfide NPs in ELISA plates and incubated at 24°C for 72 hours. Test and control wells were washed twice by cold PBS solution and centrifuged in 1400 rpm for 10 min. 100 *μ*L Annexin-V FITC solution and 100 *μ*L PI (propidium iodide) solution were added and incubated for 15 minutes at room temperature. Subsequently, cellular apoptosis in our study was detected by using Annexin-V FLUOS staining kit (Roche, Germany). The procedure was performed according to manufacturing protocol in the dark place and was evaluated FACSCalibur system. Afterwards the flow cytometry results were then analyzed using CellQuest software.

## 3. Statistical Analysis

The results of test and control groups were analyzed and compared by ANOVA statistical test (*P* ≤ 0.05) using SPSS version 15 software and Graph pad prism version 5.04. 

## 4. Results 

**Figure 1:**
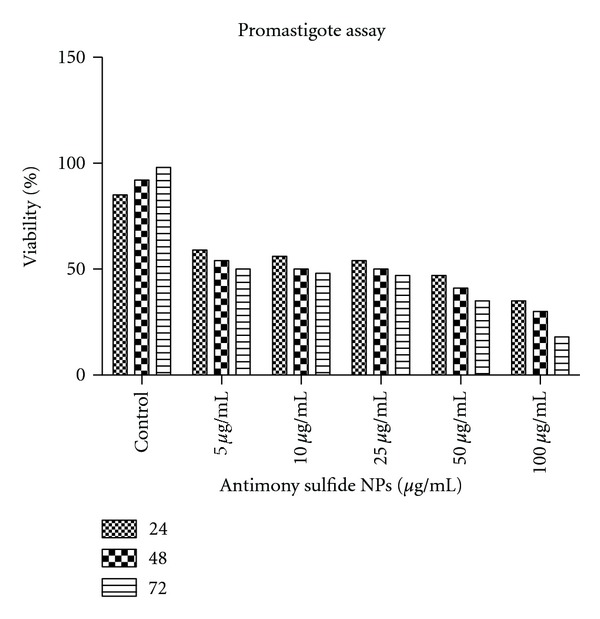
Viability of test and control groups of promastigote of *L.* *infantum* in 5 dilutions of antimony sulfide NPs during 24, 48, and 72 hours of incubation.

The results indicated the positive effectiveness of antimony sulfide NPs on proliferation of promastigote form. The cytotoxic effect of 5 dilutions of antimony sulfide NPs on promastigotes was assessed and compared with control group in Figure 1. The IC_50_ (50% inhibitory concentration) of antimony sulfide NPs on promastigotes was calculated 50 *μ*g/ml.

**Figure 2:**
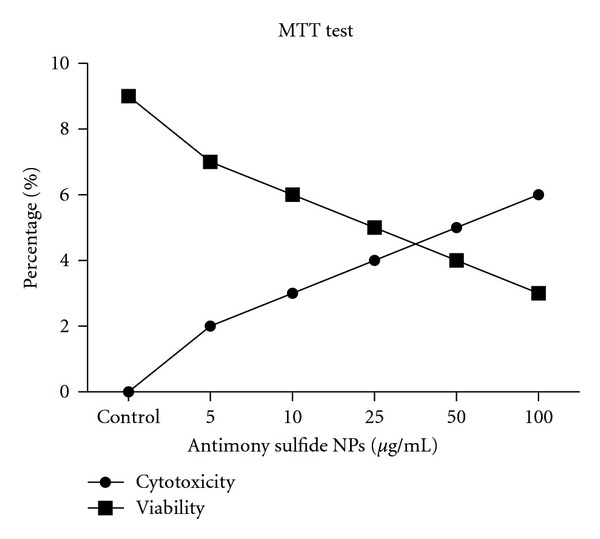
Percentage of viability of promastigotes of *Leishmania* *infantum* and cytotoxicity of 5 dilutions of antimony sulfide NPs at 72 hours by MTT method.

MTT method was used for verification results of promastigote assay. Cytotoxicity of different concentration of the drug and viability of promastigote stage of the parasite are shown in Figure 2. By increasing the concentration of antimony sulfide NPs, the cytotoxicity curve raised and the viability curve of the parasite dropped simultaneously.

**Figure 3:**
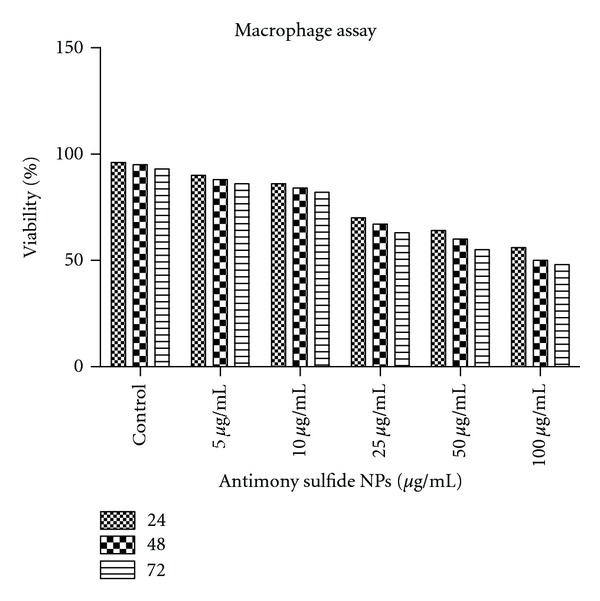
Viability of uninfected mouse macrophages in 5 dilutions of antimony sulfide NPs *in vitro* conditions during 24, 48, and 72 hours of incubation.

Cytotoxic effect of 5 dilutions of antimony sulfide NPs on uninfected splenic macrophages of BALB/c mice was compared with control cultures at 24, 48, 72 hours. The result as is shown in Figure 3, indicated that antimony sulfide NPs has low cytotoxicity effect on uninfected macrophages.

**Figure 4:**
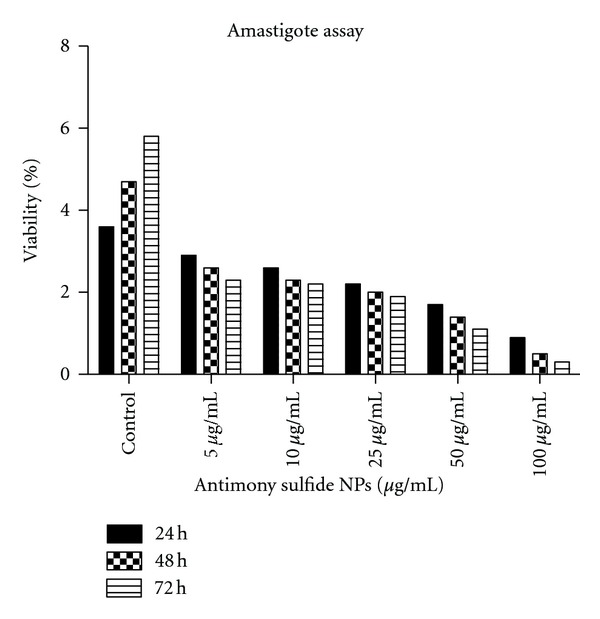
Viability of mouse macrophages contained amastigotes of *Leishmania infantum* in 5 dilutions of antimony sulfide NPs *in vitro* conditions during 24, 48, and 72 hours of incubation.

The viability of mouse macrophages contained amastigotes of *Leishmania infantum* in 5 dilutions of antimony sulfide NPs in vitro conditions during 24, 48, and 72 hours of incubation is shown in Figure 4. Moreover, the IC_50_ of antimony sulfide NPs on amastigote stage of *L. infantum* was calculated 25 *μ*g/mL.

**Figure 5:**
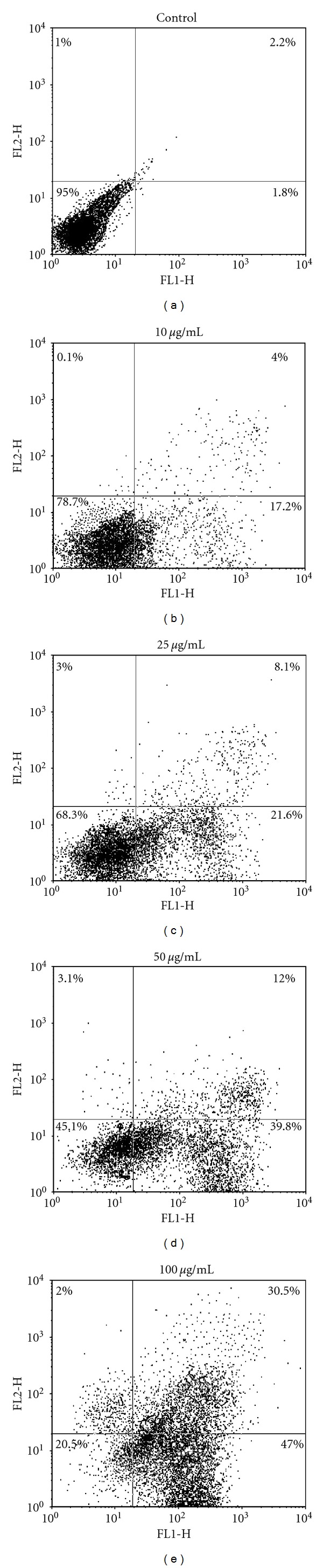
Induction of apoptosis in promastigotes of *Leishmania infantum* evaluated by flow cytometry method. (a) Most of promastigotes were alive and healthy (control group), (b) under 10 *μ*g/mL of antimony sulfide NPs, (c) under 25 *μ*g/mL of antimony sulfide NPs, (d) under 50 *μ*g/mL of antimony sulfide NPs, and (e) under 100 *μ*g/mL of antimony sulfide NPs.

The percentages of apoptotic, necrotic, and viable cells were determined by flow cytometry. The basis of 4 areas are the cells staining with Annexin-V only as apoptotic cells (lower right region), the cells staining with PI as necrotic cells (upper left region), the cells staining with both of Annexin-V and PI as late apoptotic (upper right region), and those cells that did not stain as healthy cells (lower left region). The result indicated that antimony sulfide NPs can induce apoptosis in promastigote stage of *Leishmania* at 3 of 4 concentrations (Figure 5).

## 5. Discussion 

Pentavalent antimonials including meglumine antimoniate (Glucantime) and stibogluconate sodium (Pentostam) are considered the drugs of choice for treatment of all clinical forms of leishmaniasis, for 40 years [30–36]. Despite some limitations attributed to its use due to resistance reported, high toxicity, various side effects and the high cost, they still remain the most important drugs against leishmaniasis. So, the need for new effective drugs with low toxicity and more effectiveness is critical. 

Nanotechnology can be a useful tool for synthesize new drugs against infectious diseases. Nanoparticles like emulsomes, liposomes, and nanospheres have been of great importance for drug delivery as drug carriers [37]. Among several nanoparticles implementing for treatment, liposomes are the best for evaluating the efficacy of antileishmanial activity of drugs as compared to any other parasitic disease mainly due to the fact that *Leishmania* parasite resides within the macrophages which are responsible for clearance of liposomes *in vivo* [38]. Liposomal formulation with drug has been proved to be successful against leishmaniasis. Moreover, the use of conventional liposomes with antileishmanial drugs has been proved to be associated with the reduction in their toxicity profile [37]. 

In fact macrophage surface contains receptors that recognize terminal galactose, mannose, fucose, or glucose residues of glycosides therefore sugar bearing liposomes were designed for improvement in macrophage targeting of antileishmanial agents [39]. In addition, mannose-grafted liposomal form was more efficient in transporting the drug to macrophages [40]. Furthermore, macrophages upon interaction with particulate drug delivery vehicles may act as secondary drug repository and contribute in localized delivery of the drug at the infected site [39].

In addition, polymeric particles like synthetic aliphatic polyesters (polylactic acid) PLA, polyglycolic acid, and their copolymers (PLGA, or polycaprolactone) are the primary candidates for the development of nanoparticle-based delivery system. They offer several advantages as compared with liposomes: high drug-loading capacity, long-term stability, and suitability for oral administration. Moreover, they can control the drug release. When prepared with biodegradable and biocompatible polymers, they are welltolerated [41]. They may consist of either a polymeric matrix (nano- or microspheres) or of a reservoir system (nano- or microcapsules).

In this regard, Venier-Julienne et al. used PLGA-NP for delivery of amphotericin B against *L. donovaniin vitro* [18]. The activity of pentamidine loaded poly (D, L-lactide) nanoparticles against *L. infantum* in a murine model has been investigated by Durand et al. [19]. The activity and ultrastructural localization of primaquine-loaded poly (D, L-lactide) nanoparticles in *L. donovani* infected mice has been conducted by Rodrigues et al. [20]. The antileishmanial activities of 2′,6′-dihydroxy-4′-methoxychalcone by entrapment in poly(D,L-lactide) nanoparticles has been investigated by Torres-Santos et al. [21]. Durand et al. studied the activity of pentamidine loaded methacrylate nanoparticles against *L.* *infantum* in a mouse model [22]. Gaspar et al. studied *in vitro* activity of primaquine-loaded poly(alkyl cyanoacrylate) nanoparticles against intracellular *L.* *donovani* [23]. Espuelas et al. studied *in vitro* antileishmanial activity of amphotericin B loaded in poly(epsilon-caprolactone) nanospheres [24]. 

These nanoparticles in this study were prepared in the Department of Pharmaceutical Biotechnology and Pharmaceutical Sciences Research Centre, Faculty of Pharmacy, Tehran University of medical sciences. The antimony NPs in our study were composed of sulfur and antimony atoms at ratio of 84/16 and these particles were as Sb_2_S_5_ in their cytoplasm or other internal bacterial spaces according to Bahrami et al. Other characteristics of antimony sulfide NPs in this study and its green synthetic method are present in Bahrami et al. literature [28]. In our study, instead of using any additional compound for delivery of antimony sulfide, nanoparticle form of the drug was synthesized and its effects on *Leishmania infantum* *in vitro* condition were evaluated. Our results indicated the positive effectiveness of antimony sulfide NPs on proliferation of promastigote form. In addition, the drug can induce apoptosis in promastigotes. So these particles can be useful for elimination of parasite. Surely this study was performed as preliminary work and further studies on the drug are needed.
